# Will we cure cancer by sequencing thousands of genomes?

**DOI:** 10.1186/1755-8166-6-57

**Published:** 2013-12-13

**Authors:** Joshua M Nicholson

**Affiliations:** 1 Virginia Tech, Department of Biological Sciences, 1981 Kraft Dr, Blacksburg, VA 24060, USA

**Keywords:** Cancer genome, Cancer, Karyotype, Oncogenes, Tumor suppressor genes

## Abstract

The promise to understand cancer and develop efficacious therapies by sequencing thousands of cancers has not occurred. Mutations in specific genes termed oncogenes and tumor suppressor genes are extremely heterogeneous amongst the same type of cancer as well as between cancers. They provide little selective advantage to the cancer and in functional tests have yet to be shown to be sufficient for transformation. Here I discuss the karyotyptic theory of cancer and ask if it is time for a new approach to understanding and ultimately treating cancer.

## 

“We can carry on and sequence every piece of DNA that ever existed, but I don’t think we will find any Achilles heels.”- James Watson, Cancer World 2013

## Background

By 2005 hundreds of gene mutations had been identified in individual cancers, it was unclear however, how prevalent these gene mutations were in cancers and which were specific to a certain type of cancer, if any. To answer these questions it was proposed to sequence thousands of cancers [1].

## Main text

**Figure 1:**
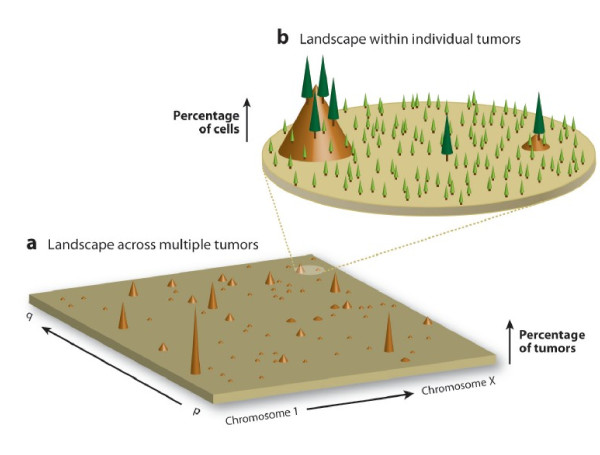
**(A) The cancer genome landscape adapted from Salk *et. al.***[9]** and Wood *et. al.***[2]**.** The cancer genome landscape illustrates that the same type of cancer does not share most mutations. The peaks correspond to how frequently a specific gene is mutated in a particular type of cancer. Large peaks, termed “mountains” indicate gene mutations that occur frequently amongst the same type of cancer while small peaks, termed “hills” indicate infrequent gene mutations. **(B)** In addition to intertumor mutational heterogeneity there exists widespread intratumor mutational heterogeneity. Within a tumor some mutations are present in the majority of single cells, depicted here as “trees”, while other mutations exist only subclonally amongst a tumor, depicted here as “seedlings”.

The first and most consistent finding from cancer sequencing studies has been that most cancers do not share the same mutations; they are so-called “hills” on the mutational landscape [2] (Figure 1). Sequencing acute myeloid leukemia (AML) from a single patient revealed 10 non-synonymous mutations in protein coding genes yet when 187 other AML patients were screened none of the same mutations were found [3]. Lung cancer sequencing showed that only 4 out of 623 genes analyzed were in more than 10% of tumors [4]. One of the most promising candidates for a cancer-specific mutation from sequencing studies, isocitrate dehydrogenase (*IDH1*) [5], was found to be mutated in only 12% of glioblastoma multiforme [6] and 8% of AML [7]. In addition to the intertumor mutational heterogeneity there exists widespread intratumor mutational heterogeneity with the majority of mutations not shared across different regions of the same tumor [8] (Figure 1).

Consistent with these findings the effects of mutations in oncogenes and tumor suppressors are now thought to be small. Indeed, modeling the effects of mutations in oncogenes and tumor suppressor genes indicate they provide very little selective advantage to the cancer, a “surprisingly small” 0.4% [10]. Functional tests of oncogenes and tumor suppressor genes further call into question the role these mutations play in carcinogenesis. Initial tests of oncogenes and tumor suppressor genes as transforming agents were largely performed in a mouse cell line NIH/3T3, which is considered a model for normal cells. Yet, this line has ~70 chromosomes instead of 40 [11], becomes transformed with a mere change in culturing conditions [12], can lose the postulated initiating oncogenes without change in carcinogenicity [13], and above all is by itself tumorigenic [14,15]. Despite these well-documented caveats NIH/3T3 cells are *still* used for transformation assays by scientists [16]. It may be argued that other lines, such as the human embryonic kidney 293 cell line (HEK293) and mammary epithelial cell line MCF10A have been successfully used to identify oncogenes and tumor suppressor genes as transforming agents. These lines, however, are prone to transformation and are known to have abnormal karyotypes [17]. Indeed, transformation by oncogenes, such as ras, are typically only successful when they are activated by viral promoters reaching levels of expression hundreds of fold higher than what is ever actually seen in cancer and in which the karyotypes are altered [18,19]. Even then, only after long latencies of many cell generations will minute fractions of transfected cells ever transform into cancer [20-22]. The consistent finding that mutations in cancer are largely heterogeneous has been hard to reconcile with the idea that only mutations in specific genes are the cause of cancer—as some sequencing studies suggest [23].

## An alternative theory of cancer

**Figure 2:**
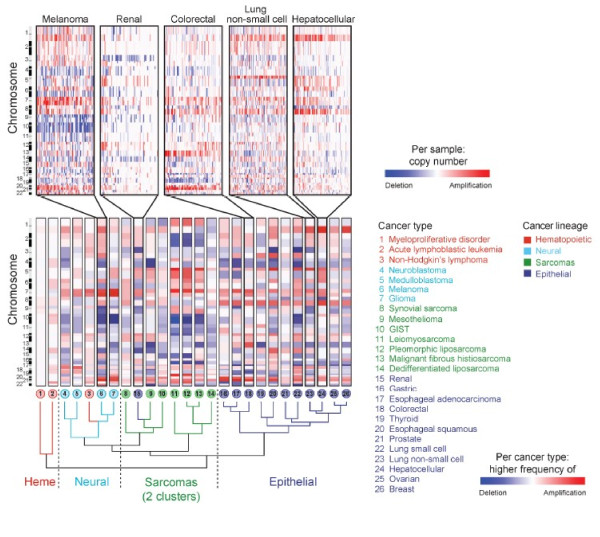
**Chromosome arm-level somatic copy-number alterations (SCNAs) identified in 26 different cancer types by Beroukhim *et. al.***[27]**.** SCNAs are displayed across all autosomes where red and blue indicate gain and loss respectively. Different cancer types are organized by unsupervised hierarchical clustering.

All cancers display at least one numerical or structural chromosome aberration [24,25]. Analysis of chromosomal gains and losses across many cancers have revealed predominant karyotypic patterns in cancer, to the extent that cancer types can be clustered based on karyotypes [26-29] (Figure 2). The non-random gains and losses of chromosomes illustrate the importance of aneuploidy in carcinogenesis. These observations, along with the fact that aneuploidy is a prognostic indicator of patient survival [24], have led to the proposal that aneuploidy causes cancer [20,21,30-36]. Still, such karyotypic patterns and aneuploidy in general have been proposed to merely reflect the gain and loss of cancer genes [27,37,38]. To test this hypothesis I compared the frequency a chromosome is gained or lost to the number of cancer genes per chromosome. If cancer karyotypes were selected for solely on cancer genes we would expect the number of cancer genes to relate to the frequency a chromosome is gained or lost.

**Figure 3:**
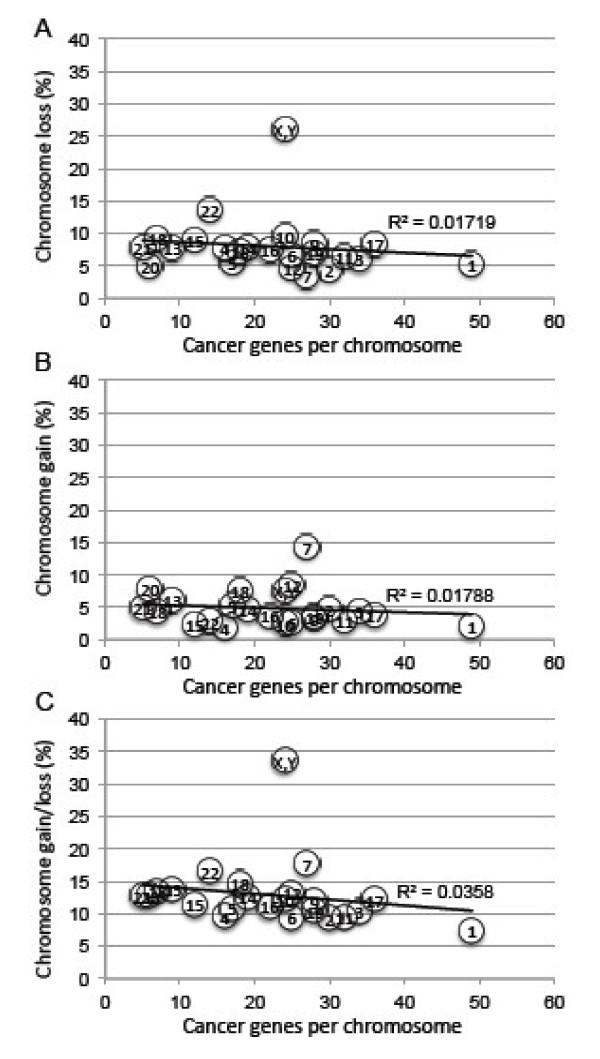
**The frequency a chromosome is gained or lost in cancer does not merely reflect the gain and loss of cancer genes.** The numbers in circles are human chromosome numbers. The number of cancer genes per chromosome is compared to the frequency of chromosome gain **(A)**, chromosome loss **(B)**, and chromosome gain and loss **(C)** in 19,003 cancers as reported in The Mitelman Database [25].

The cancer gene census currently lists a total of 507 cancer genes [39]. Analysis of 19,003 solid cancers reported in the Mitelman Database revealed 35,021 chromosome losses and 21,268 gains [25,38]. Organizing these cancer genes by chromosome and comparing them to how frequently they are lost or gained in cancer shows that there is no relationship between the number of cancer genes on a chromosome and the frequency that chromosome is gained or lost (Figure 3). The gain or loss of a single chromosome is thus not simply the gain or loss of a cancer gene. Indeed, a single trisomy induces complex gene expression changes across the entire genome [40,41]. Such karyotypic patterns then must represent the selection of the entire karyotype, given that a gain or loss of a chromosome affects the entire genome.

## Conclusion

If the karyotype is central to carcinogenesis and not individual genes, will we cure cancer by sequencing thousands of genomes or is it time for a new approach?
